# Serotonin immunoreactive interneurons in the brain of the Remipedia: new insights into the phylogenetic affinities of an enigmatic crustacean taxon

**DOI:** 10.1186/1471-2148-12-168

**Published:** 2012-09-05

**Authors:** Torben Stemme, Thomas M Iliffe, Gerd Bicker, Steffen Harzsch, Stefan Koenemann

**Affiliations:** 1 Division of Cell Biology, University of Veterinary Medicine Hannover, Bischofsholer Damm 15, Hannover, 30173, Germany; 2 Department of Marine Biology, Texas A&M University, 200 Seawolf Pkwy, Galveston, TX, 77553, USA; 3 Department of Cytology and Evolutionary Biology, Institute of Zoology, Ernst-Moritz-Arndt-University of Greifswald, Soldmannstraße 23, Greifswald, 17487, Germany; 4 Montessori Bildungshaus Hannover, Bonner Straße 10, Hannover, 30173, Germany

**Keywords:** Arthropoda, Comparative neuroanatomy, Sister group relationship, Immunocytochemistry, Olfactory glomeruli, Central complex, Hemiellipsoid bodies, Olfactory globular tracts, *Speleonectes*, *Godzilliognomus*

## Abstract

**Background:**

Remipedia, a group of homonomously segmented, cave-dwelling, eyeless arthropods have been regarded as basal crustaceans in most early morphological and taxonomic studies. However, molecular sequence information together with the discovery of a highly differentiated brain led to a reconsideration of their phylogenetic position. Various conflicting hypotheses have been proposed including the claim for a basal position of Remipedia up to a close relationship with Malacostraca or Hexapoda. To provide new morphological characters that may allow phylogenetic insights, we have analyzed the architecture of the remipede brain in more detail using immunocytochemistry (serotonin, acetylated α-tubulin, synapsin) combined with confocal laser-scanning microscopy and image reconstruction techniques. This approach allows for a comprehensive neuroanatomical comparison with other crustacean and hexapod taxa.

**Results:**

The dominant structures of the brain are the deutocerebral olfactory neuropils, which are linked by the olfactory globular tracts to the protocerebral hemiellipsoid bodies. The olfactory globular tracts form a characteristic chiasm in the center of the brain. In *Speleonectes tulumensis*, each brain hemisphere contains about 120 serotonin immunoreactive neurons, which are distributed in distinct cell groups supplying fine, profusely branching neurites to 16 neuropilar domains. The olfactory neuropil comprises more than 300 spherical olfactory glomeruli arranged in sublobes. Eight serotonin immunoreactive neurons homogeneously innervate the olfactory glomeruli. In the protocerebrum, serotonin immunoreactivity revealed several structures, which, based on their position and connectivity resemble a central complex comprising a central body, a protocerebral bridge, W-, X-, Y-, Z-tracts, and lateral accessory lobes.

**Conclusions:**

The brain of Remipedia shows several plesiomorphic features shared with other Mandibulata, such as deutocerebral olfactory neuropils with a glomerular organization, innervations by serotonin immunoreactive interneurons, and connections to protocerebral neuropils. Also, we provided tentative evidence for W-, X-, Y-, Z-tracts in the remipedian central complex like in the brain of Malacostraca, and Hexapoda. Furthermore, Remipedia display several synapomorphies with Malacostraca supporting a sister group relationship between both taxa. These homologies include a chiasm of the olfactory globular tract, which connects the olfactory neuropils with the lateral protocerebrum and the presence of hemiellipsoid bodies. Even though a growing number of molecular investigations unites Remipedia and Cephalocarida, our neuroanatomical comparison does not provide support for such a sister group relationship.

## Background

**Figure 1:**
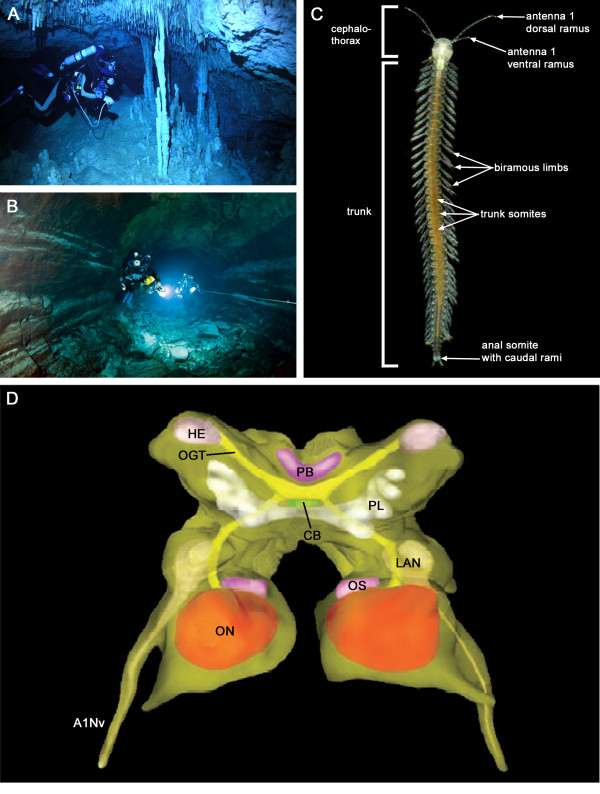
**Habitat, external morphology and brain architecture of Remipedia.** (**A, B**) Photographs of the interior of anchialine caves give an impression of the difficulties in collecting Remipedia. (**A**) Cenote Carwash near Tulum, Mexico is the type locality for the remipede *Speleonectes tulumensis* (photo by Joerg Hess). (**B**) Tunel de Atlantida, a volcanic cave in Lanzarote, Canary Islands, contains two species of *Speleonectes* (photo by Jill Heinerth). (**C**) Photograph of *Speleonectes tanumekes*. The body of Remipedia can be divided into two main regions, a cephalothorax composed of six appendage-bearing somites including biramous first antennae and a homonomous trunk with numerous segments, each equipped with a pair of biramous limbs (modified from [2]; Photograph courtesy of J. van der Ham). (**D**) Modified 3D reconstruction of the general brain anatomy of Remipedia adopted from [15]. Abbreviations: A1Nv: antenna 1 nerve; CB: central body; HE: hemiellipsoid body; LAN: lateral antenna 1 neuropil; OGT: olfactory globular tract; ON: olfactory neuropil; OS: olfactory satellite neuropil; PB: protocerebral bridge; PL: protocerebral sublobes.

Remipedia are remarkable, eyeless crustaceans that are characterized by uniformly organized metameric body segments. The first living specimens of Remipedia were collected 1979 in the Bahamas Archipelago [1]. To date, three extant families (Godzilliidae, Speleonectidae and Micropacteridae) including 26 species of Remipedia have been described. The habitat of Remipedia is generally confined to anchialine cave systems in tropic and subtropic regions (Figure 1A, B). Remipedia are mainly distributed in the Caribbean Sea, but isolated populations appear on Lanzarote (Spain) and Western Australia [2].

They are pale and eyeless animals composed of two main body regions: a cephalothorax consisting of the cephalon fused with the first trunk segment bearing the maxillipeds and a long homonomous trunk (Figure 1C). Whereas all other extant groups of Crustacea feature a trunk division in at least two tagmata [3], Remipedia lack trunk tagmosis. Due to the metameric trunk and other characters that are considered plesiomorphic for Crustacea (e.g., paddle-like appendages and a cephalic shield) [4], Remipedia were regarded as basal crustaceans in most early morphological comparisons, and suggested to be the sister group of all other Crustacea [4-7]. Subsequent analyses suggested a sister group relationship to various crustacean taxa, e.g., Cirripedia, Ostracoda, Cephalocarida and “maxillopodan” taxa (review [8] and references therein). Additionally, a sister group relationship of Remipedia to Diplura [9] or Collembola [10,11] has been proposed. In the last decade, several morphological, developmental and molecular phylogenetic analyses suggested that Remipedia represent a derived rather than an ancestral taxon and may be close allies of Malacostraca or Hexapoda. Specifically, an early analysis of morphological characters provided the first evidence for a close relationship between Remipedia and Hexapoda [12]. Developmental comparisons showed similarities between the larvae of Remipedia and those of Malacostraca [13,14]. Based on their complex brain architecture, Fanenbruck et al. [15,16] suggested that Remipedia are closely related to Malacostraca and Hexapoda. Concerning molecular studies, comparisons of hemocyanin sequences [17] and molecular sequence analyses [9,18-20] added support to this classification. Most recently, Regier et al. [19] recovered a new clade “Xenocarida” consisting of Remipedia and Cephalocarida as sister taxa, which together with Hexapoda was suggested to form the taxon “Miracrustacea”. In summary, in most recent phylogenetic approaches, Remipedia are not regarded as basal Crustacea anymore, but a defined sister taxon of Remipedia is still elusive.

In arthropods, chemical neuroanatomy has proven to be a useful approach for obtaining independent, new phylogenetic characters, in addition to external morphology and molecular sequence information [21-24]. The arthropod nervous system has been the target of an increasing number of comparative neuroanatomical studies, many of which focussed on non-model species chosen according to phylogenetic aspects [24-27]. A wide variety of crustacean nervous systems both of Malacostraca and non-malacostracans have been investigated by combining immunocytochemistry, confocal laser-scanning microscopy and computer-based 3D-reconstruction [28-33].

The Crustacea were suggested to comprise five major taxa: Malacostraca, Branchiopoda, “Maxillopoda”, Cephalocarida and Remipedia [5]. An investigation of the brain with advanced neuroanatomical techniques is lacking only for Remipedia. One reason for this gap in our knowledge certainly is their late discovery, while another is the difficulty of collecting specimens from inaccessible cave systems (Figure 1A, B). The first neuroanatomical studies on Remipedia were based on conventional serial sectioning and light microscopy [15,16] and provided an anatomical atlas of the remipede brain (Figure 1D). Despite the absence of eyes and optic lobes, the well-differentiated brain led the authors to suggest a derived rather than a basal position of Remipedia within Crustacea and a close affinity to Malacostraca and Hexapoda. This claim was based on the architecture of the central complex, the connectivity between proto- and deutocerebrum by a distinct neurite bundle (called olfactory globular tract in Crustacea and antennocerebral tract in Insecta) and the organization of the olfactory system [15,16]. As in Malacostraca and Insecta, the input from antenna 1 in Remipedia is not only chemosensory, but has most likely also mechanosensory components that are processed in separate neuropils of the brain. The remipede olfactory neuropils are linked to the hemiellipsoid bodies of the protocerebrum by the olfactory globular tract, the two arms of which cross at the midline thus forming a chiasm. Likewise, the antennal lobes of insects are connected to the mushroom bodies of the protocerebrum by one or several antennocerebral tracts, but the arms of these tracts do not form a chiasm. However, the previous neuronanatomical structures in Remipedia show characteristic differences that question their homology to Malacostraca and Hexapoda (e.g. the absence of W-, X-, Y-, Z-tracts between the protocerebral bridge and the central body in Remipedia). Therefore, to extend our knowledge on remipede neuroanatomy, we use transmitter immunocytochemistry in the present account, which provides information on the morphology and specific biochemical pathways in individual neurons. We apply immunofluorescence and confocal laser-scanning microscopy to resolve the projection pattern of identifiable groups of neurons in the remipede brain. In this first account of the chemical neuroanatomy of Remipedia, we combined immunolocalization of the biogenic amine serotonin with fluorescence labeling of neuropil regions, neurite tracts, and soma clusters. Neuropil regions were stained using an antibody against synapsin, neurite tracts were identified by acetylated α-tubulin immunocytochemistry, and soma clusters by counterstaining of nuclei with 4^′^6-diamidine-2-phenylidole-dihydrochloride (DAPI). Apart from a description of general brain anatomy, we focus on prominent serotonin immunoreactive innervation of the deutocerebral olfactory pathway and the protocerebral unpaired midline neuropil. This allows detailed comparisons to related arthropod taxa.

## Results

### General architecture of the remipede brain

Despite the absence of a visual system, the remipede brain comprises a protocerebrum, deutocerebrum, and tritocerebrum [15,16]. The tritocerebrum and ventral nerve cord are orientated almost in line with the body axis. However, the deuto- and protocerebrum are rotated out of the body axis for approximately 180° backwards, which results in an inverted neuraxis with respect to the body axis (see Figure 1 in [15] and Figures 3-4 in [16] for sideviews). In our study, directional descriptions of anatomical structures refer to the body axis. Thus, anterior of the body axis points towards the bottom in each panel so that the neuraxis of proto- and deutocerebrum faces upwards. This convention facilitates comparisons to the many illustrations published on other arthropod nervous systems.

#### Protocerebrum

**Figure 2:**
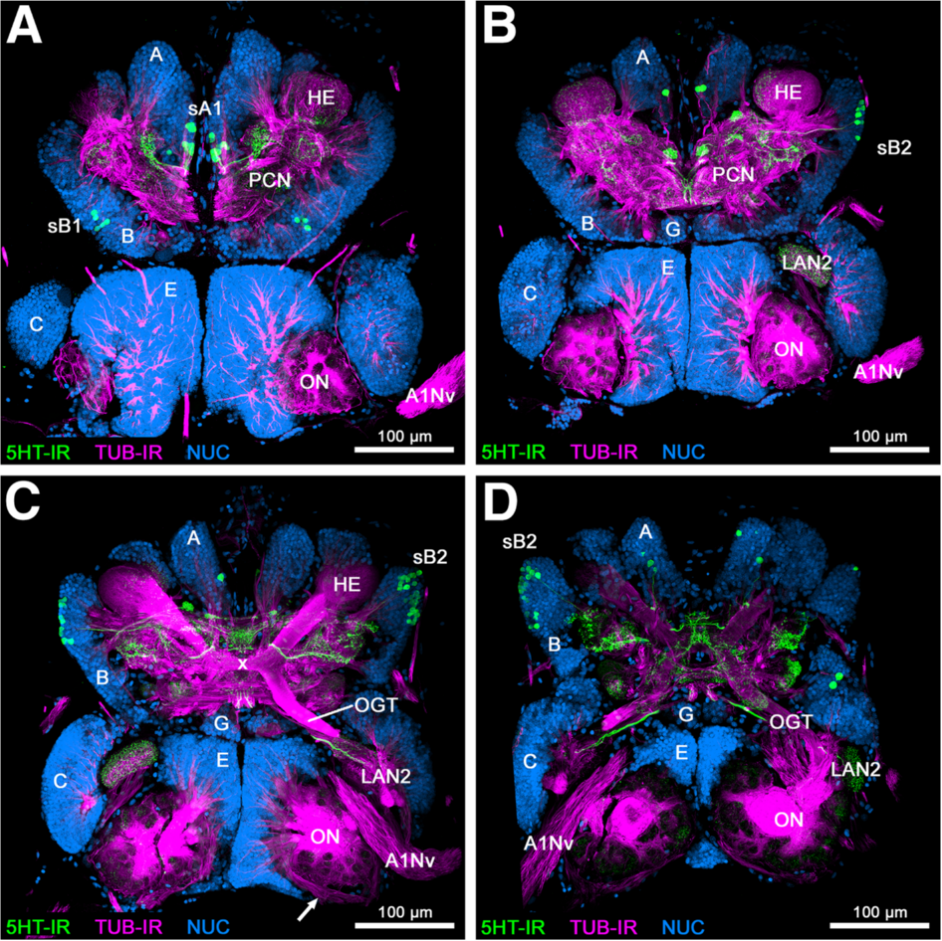
**1**^**st**^**part of a series of horizontal vibratome sections of the brain of*Speleonectes tulumensis*.** (**A-D**) Dorsal to ventral series of vibratome sections (50 μm) through the brain of *Speleonectes tulumensis* triple labeled for serotonin immunoreactivity (5HT-IR, green), acetylated α-tubulin immunoreactivity (TUB-IR, magenta) and nuclear marker (NUC, blue). Maximum projections consisting of 32 to 44 confocal laser-scans per slice. Arrow in (**C**) indicates a neurite bundle that comprises the axons from the olfactory receptor neurons. Abbreviations: capital letters A, B, C, E, G: soma clusters; A1Nv: antenna 1 nerve; HE: hemiellipsoid body; LAN2: lateral antenna 1 neuropil 2; OGT: olfactory globular tract; ON: olfactory neuropil; PCN: protocerebral neuropil; sA1, sB1, sB2: 5HT-ir cell groups; x: chiasm of the OGT.

**Figure 3:**
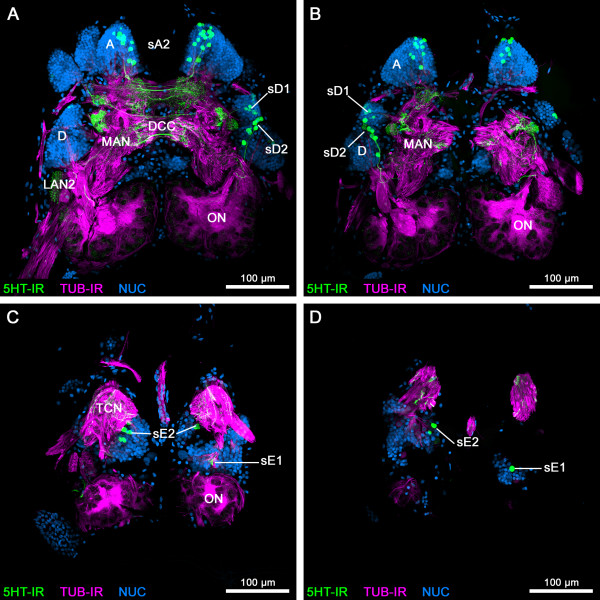
**2**^**nd**^**part of a series of horizontal vibratome sections of the brain of*Speleonectes tulumensis*.** (**A-D**) Dorsal to ventral series of vibratome sections (50 μm) through the brain of *Speleonectes tulumensis* triple labeled for serotonin immunoreactivity (5HT-IR, green), acetylated α-tubulin immunoreactivity (TUB-IR, magenta) and nuclear marker (NUC, blue). Maximum projections consisting of 31 to 41 confocal laser-scans per slice. Abbreviations: capital letters A, D: soma clusters; DCC: deutocerebral commissural neurites; LAN2: lateral antenna 1 neuropil 2; MAN: medial antenna 1 neuropil; ON: olfactory neuropil; sA2, sD1, sD2, sE1, sE2: 5HT-ir cell groups; TCN: tritocerebral neuropil.

**Figure 4:**
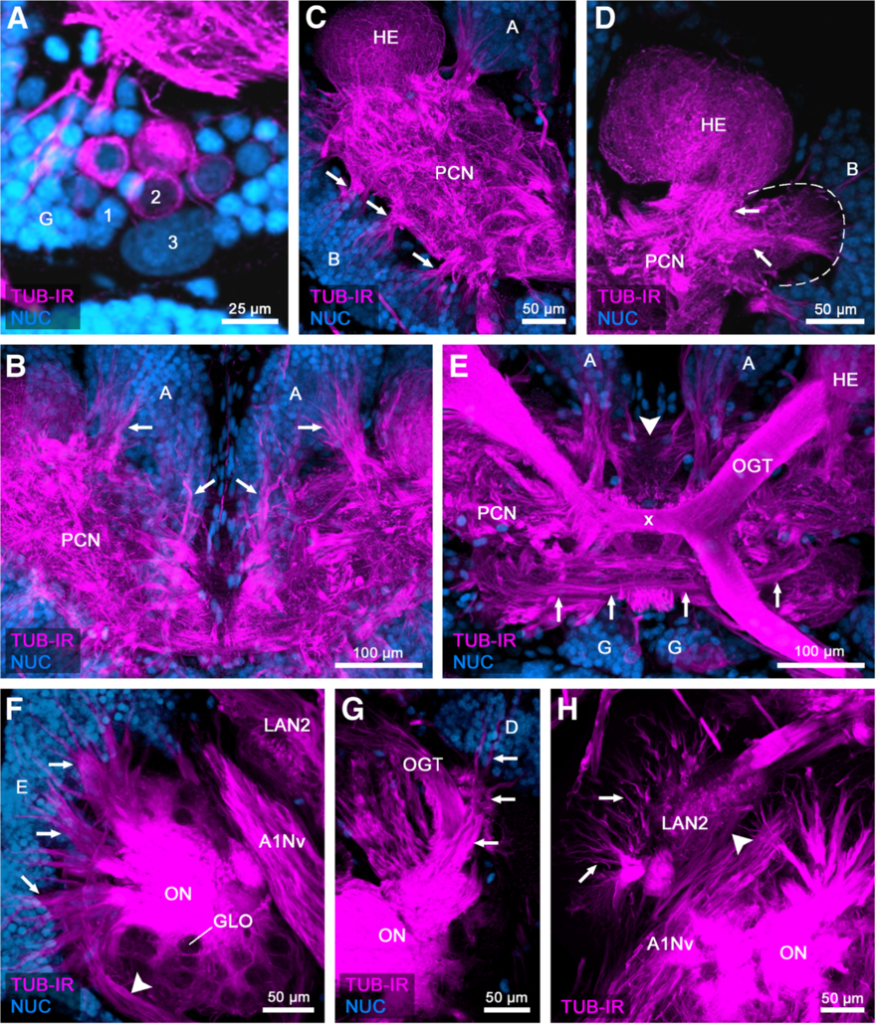
**General anatomy of the brain of* Speleonectes tulumensis *.** (**A**-**H**) Dorsal view of confocal laser-scans from horizontal vibratome sections (50 μm) labeled for acetylated α-tubulin immunoreactivity (TUB-IR, magenta) and nuclear marker (NUC, blue) in higher magnification as Figures 2 and 3. (**A**) Close-up of the three types (1–3) of somata in the remipede brain. (**B**) Neurites from soma cluster A extend anteriorly into the protocerebral neuropil (PCN) (arrows). (**C**) The PCN is innervated by several neurite bundles (arrows) from soma cluster B. (**D**) The hemiellipsoid body (HE) is connected by a neurite bundle (arrows) to a small neuropilar structure in lateral position (dashed line). (**E**) The olfactory globular tract (OGT) connects the olfactory neuropil (ON; not in focus) with the HE and forms a chiasm (x) in the center of the protocerebrum. Anterior to the chiasm, several commissural neurite bundles link structures of the PCN from both hemispheres (arrows). Furthermore, an unpaired midline neuropil is positioned posteriorly to the chiasm of the OGT (arrowhead). (**F**) Neurites from somata in cluster E enter the core of the ONs (arrows). The ONs are innervated by a neurite bundle, which comprises the axons from the olfactory receptor neurons (arrowhead). (**G**) Projections from soma cluster D travel anteriorly in parallel to the OGT and enter the ONs (arrows). (**H**) The lateral antenna 1 neuropil 2 (LAN2) is innervated by neurites of soma cluster C (arrows). Furthermore, one branch of the antenna 1 nerve (A1Nv) targets the LAN2 (arrowhead). Abbreviations: numbers 1–3: somata types; capital letters A, B, D, E, G: soma clusters; A1Nv: antenna 1 nerve; GLO: olfactory glomerulus; HE: hemiellipsoid body; LAN2: lateral antenna 1 neuropil 2; OGT: olfactory globular tract; ON: olfactory neuropil; PCN: protocerebral neuropil; x: chiasm of the OGT.

The protocerebral neuropil is surrounded by a cortex of neuronal somata, which can be divided into distinct bilateral soma clusters, termed A, B and G (Figures 2 and 3) [16]. Based on nuclear diameter, three types of somata can be distinguished in the remipede brain (Figure 4A). As described by Fanenbruck and Harzsch [16], several neurons with slightly larger nuclei (nuclear diameter ~13 μm) in comparison to most other neurons (nuclear diameter ~9 μm) are present in all soma clusters. These larger nuclei are surrounded by a wide cytoplasmic belt [16], as revealed by the acetylated α-tubulin labeling. In addition, we found a few cells with conspicuous ovoid giant nuclei (~30 μm at maximal expansion) in all soma clusters (Figure 4A).

The somata of cluster A cover the protocerebrum posteromedially and extend their neurites into the neuropil (Figures 2, 3 and 4B). Laterally, the protocerebral neuropil receives several neurite bundles from somata in cluster B (Figures 2A and 4C). A third soma cluster G is situated anteromedially to the protocerebrum. Neurites of these cells extend posteriorly into the protocerebral neuropil (Figure 2B).

**Figure 5:**
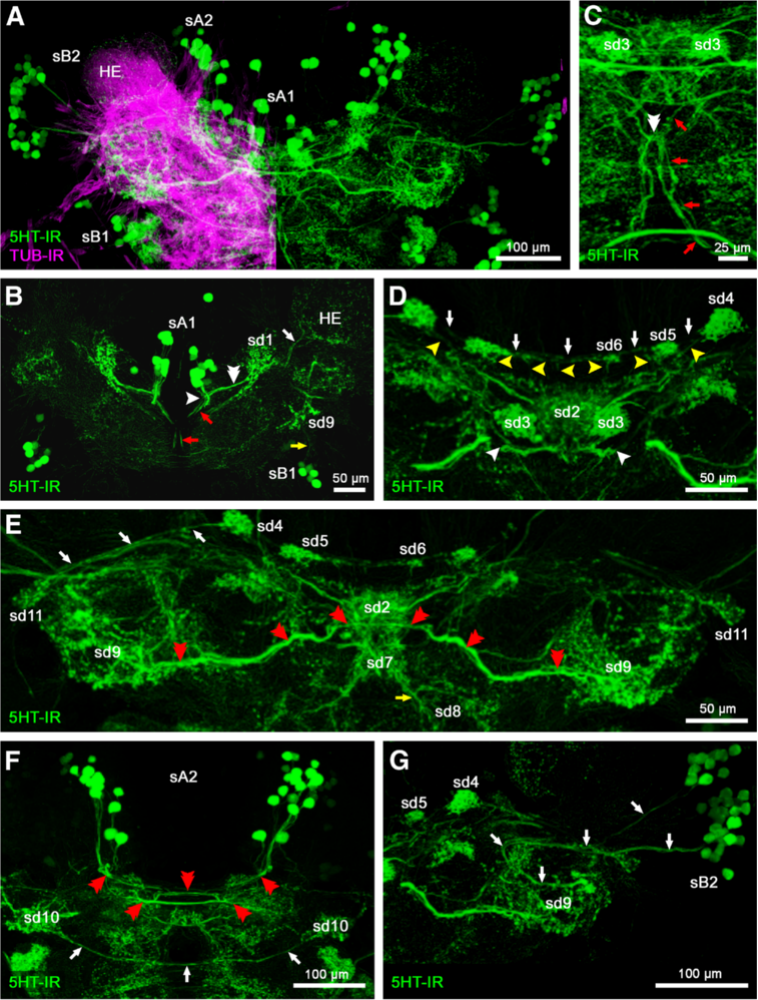
**Serotonin immunoreactivity in the protocerebrum of* Speleonectes tulumensis ***. (**A**-**G**) Dorsal view of confocal laser-scans of horizontal vibratome sections (50 μm). (**A**) Overview of serotonin immunoreactivity (5HT-IR, green) showing the distribution of 5HT-ir cells (sA1, sA2, sB1, sB2). Left brain hemisphere double labeled for acetylated α-tubulin immunoreactivity (TUB-IR, magenta), revealing the position of the hemiellipsoid body (HE). (**B**) Neurites of sA1 project anteriorly forming a convergence (arrowhead) with neurites from the 5HT-ir domain sd1 (double arrowhead). One branch leaves this convergence anteriorly to more posterior regions of the nervous system (red arrows in B and C), decussating in the center of the brain (double arrowhead in C). Cluster sB1 projects posteriorly into sd9 (yellow arrow in B). (**D**) Another branch of the convergence in (B) runs medially into a decussation and innervates sd2 (arrowheads). Sd2 is associated to spherical sd3 and sd4-6 (yellow arrowheads). The domains sd4-6 are interconnected by faintly stained longitudinal neurites that decussate at the midline (arrows). (**E**) Neurites from sd2 run anteriorly to another unpaired midline neuropil (sd7) and further anterior to the paired sd8 (yellow arrow). Additionally, sd4 is interconnected to sd11 (arrows). The lateral positioned sd9 are connected by a heavily labeled commissure (red double arrowheads) composed of neurites of cluster sA2 (see F). (**F**) Cluster sA2 projects anteriorly into the neuropil forming numerous commissures. The most noticeable one passes sd2 ventrally projecting into the vesicular sd9 (red double arrowheads in E and F). The domain sd9 is interconnected to the more dorsally positioned sd10. Both parts of sd10 are connected via a thin commissure (arrows in F). (**G**) Projections from cluster sB2 enter the protocerebrum, deflect in a conspicuous loop, grow again laterally and end in the vesicular sd9 (arrows). Abbreviations: HE: hemiellipsoid body; sA1, sA2, sB1, sB2: 5HT-ir cell groups; sd1-11: 5HT-ir domains.

The protocerebrum of the investigated remipede species comprises the following prominent structures: the paired hemiellipsoid bodies (HEs), the olfactory globular tracts (OGTs) and an unpaired midline neuropil, resembling a central body (Figures 2, 3, 4 and 5) [15,16]. Labeling against acetylated α-tubulin clearly identifies the spherical HEs (Figures 2A, B and 4C, D) which are targeted by the OGTs (Figures 2C and 4E). The internal structure of the HEs consists of fine dense processes that are not arranged in any distinct layers (Figure 4D). The paired branches of the OGT form a characteristic chiasm in the center of the protocerebrum (Figures 2C and 4E). The HEs are connected by a conspicuous neurite bundle to a small lateral structure of the protocerebrum (Figure 4D). Several commissural neurite bundles anterior to the chiasm of the OGT link the protocerebral structures (Figure 4E). A small unpaired medial neuropil is positioned posteriorly near the chiasm of the OGT (Figure 4E). Due to the medial position in the center of the protocerebrum, we suggest that this unpaired midline neuropil corresponds to the central body. Labeling against acetylated α-tubulin and synapsin does not resolve whether the unpaired midline neuropil is subdivided into a protocerebral bridge and a central body, or how these structures are associated to the lateral protocerebral neuropils. Nevertheless, labeling of serotonin reveals subcompartments within the unpaired midline neuropil and elucidates its connectivity to the lateral accessory lobe (see below).

#### Deutocerebrum

**Figure 6:**
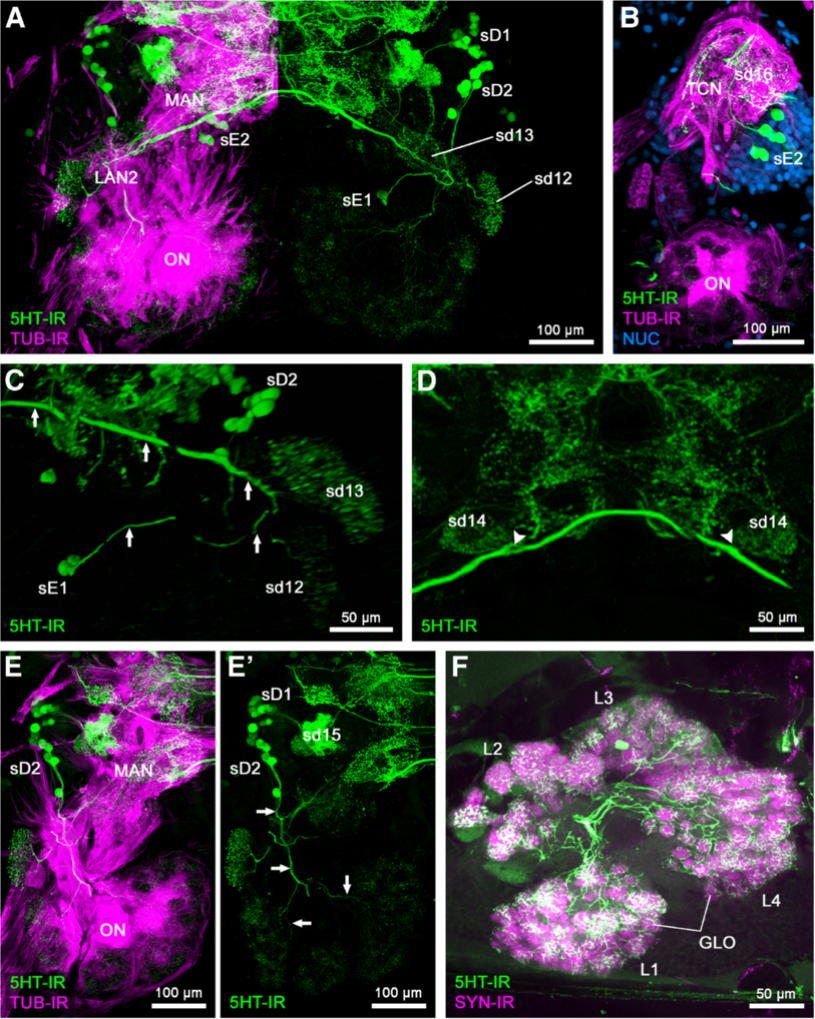
**Serotonin immunoreactivity in the deuto- and tritocerebrum of* Speleonectes tulumensis ***. (**A**, **B**, **D**, **E**, **E**’) Dorsal view on confocal laser-scans of horizontal vibratome sections (50 μm). (C) Amira 3D virtual stack. (**F**) Maximum projection of vibratome cross section (100 μm). (**A**) Overview of the serotonin immunoreactivity (5HT-IR, green) in the deutocerebrum showing the distribution of 5HT-ir cells (sD1, sD2, sE1, sE2). Left brain hemisphere double labeled for acetylated α-tubulin immunoreactivity (TUB-IR, magenta). (B) Neurites of cluster sE2 extend posteriorly into sd16 which is situated in the tritocerebral neuropil (TCN). Triple labeling for 5HT-IR (green), TUB-IR (magenta) and nuclear marker (NUC, blue). (**C**) The two neurons from sE1 project their neurites in a loop-like manner dorsolaterally innervating two 5HT-ir domains of the LAN2 (sd12, sd13; arrows). (**D**) The main neurite bundle of sE1 continues ventromedially crossing the midline and forming a decussation with its counterpart from the other brain hemisphere. Near the midline, the sd14, which seems to be associated to the MAN, is innervated (arrowheads). (**E**, **E**’) Projections of sD2 extend posterior into the core of the olfactory neuropils (ONs), sending neurites parallel to the OGT (arrows). From the core, neurites diverge and innervate all olfactory glomeruli in the different sublobes. The cell group sD1 innervates the sd15 situated laterally in the deutocerebral neuropil. (**F**) Close-up of the innervation of sublobes and olfactory glomeruli by 5HT-ir neurites. The ONs can be divided into at least for sublobes (L1-4). Double labeling for 5HT-IR and synapsin immunoreactivity (SYN-IR, magenta). Abbreviations: GLO: olfactory glomerulus; L1-4: olfactory sublobes 1–4; LAN2: lateral antenna 1 neuropil 2; MAN: medial antenna 1 neuropil; ON: olfactory neuropil; sD1, sD2, sE1, sE2: 5HT-ir cell groups; sd12-16; 5HT-ir domains; TCN: tritocerebral neuropil.

The deutocerebrum comprises the following structures: the paired olfactory neuropils (ONs), the paired lateral antenna 1 neuropils 1 and 2 (LAN1 and LAN2) and the unpaired medial antenna 1 neuropil (MAN) (Figures 2, 3, 4 and 6) (see [16] for comparison). The ONs are the first synaptic relay stations of the chemosensory afferents from antenna 1 in the remipede brain. Labeling against acetylated α-tubulin reveals a thick neurite bundle that approaches the ONs posterolaterally, surrounding and innervating these neuropils (Figures 2C and 4F). Although the origin of this neurite bundle could not be traced in our preparations a comparison to earlier accounts on the remipede brain [15,16] shows that this neurite bundle most likely is a nerve from antenna 1 and comprises the axons from the olfactory sensory neurons that are associated with the aesthetascs of antenna 1. The ONs contain spherical olfactory glomeruli that express strong synapsin immunoreactivity (Figure 6F). We counted 363 and 393 olfactory glomeruli in the ONs of one specimen of *Speleonectes tulumensis*. The olfactory glomeruli are arranged in sublobes in a cauliflower-like manner around a core of diverging neurite bundles (Figures 2, 3 and 4F). We could resolve at least four sublobes within the ONs of *S. tulumensis* (Figure 6F). The ONs are surrounded dorsomedially by the soma cluster E (Figure 2) [16]. Projections of these neurons enter the core of the ONs (Figure 4F). In addition, neurites from soma cluster D target the ONs, traveling in parallel to the OGTs (Figures 3A, B and 4G).

The antenna 1 nerve enters the brain anterolaterally and bifurcates into two branches: one targets the LAN2, the other one the LAN1 (Figures 2, 3 and 4H). Neurites of the soma cluster C and E are associated with the LAN2, which seems to be subdivided into several compartments, whereas the LAN1 is invaded by the projections of cells from the soma cluster D. The MAN forms a diffuse neuropilar block together with the LAN1, and also receives neurites from soma cluster D. Commissural neurite bundles connect both hemispheres of the deutocerebrum (Figure 3A).

#### Tritocerebrum

The tritocerebrum is associated with the nerve of antenna 2 and the tritocerebral neuropil is innervated by soma cluster F (Figure 3C) [15,16]. In our preparations, only the most dorsal part of the tritocerebrum can be observed, therefore our description is unfortunately incomplete. More detailed information concerning the tritocerebrum of Remipedia can be found in the brain atlas by Fanenbruck and Harzsch [16].

### Distribution of serotonin immunoreactive neurons in *Speleonectes tulumensis*

**Figure 7:**
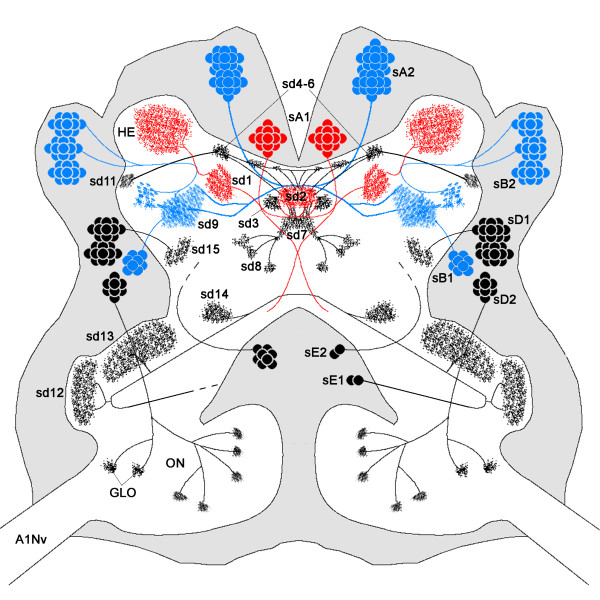
**Schematic drawing of the serotonin immunoreactivity in the brain of* Speleonectes tulumensis. *** In the left hemisphere, 117 5HT-ir neurons were detected; in the right hemisphere, 116. These neurons are arranged in distinct cell groups (sA1, sA2, sB1, sB2, sD1, sD2, sE1, sE2) within the surrounding neuronal soma clusters (grey). Neurite bundles of these 5HT-ir neurons extend into the neuropil (white) and innervate distinct neuropilar domains (sd1-9; sd11-15) in different brain areas. In the olfactory lobes (ONs), only few olfactory glomeruli (GLOs) are exemplified. Projections from protocerebral cell groups are colored in red (sA1) and blue (sA2, sB1, sB2) for better discrimination between projections of these neurons. For the same reason, the 5HT-ir domains sd10 and sd16 are omitted. Abbreviations: A1Nv: antenna 1 nerve; GLO: olfactory glomerulus; HE: hemiellipsoid body; ON: olfactory neuropil; sA1, sA2, sB1, sB2, sD1, sD2, sE1, sE2: 5HT-ir cell groups; sd1-9, sd11-15: 5HT-ir domains.

To initiate an investigation of the chemical neuroanatomy of the remipede brain, we focussed an immunofluorescence study on the localization of serotonin immunoreactivity (5HT-IR). In one specimen, we detected 116 and 117 serotonin immunoreactive (5HT-ir) somata in the brain hemispheres, which are arranged in distinct cell groups. The neurites of these 5HT-ir neurons extend into the neuropil, branching out in most parts of the brain. However, the distribution of immunoreactive neurites is by no means homogeneous, and we could resolve some characteristically dense, fluorescent neuropilar domains in different brain areas. Generally, all prominent structures of the remipede brain contain 5HT-IR (ONs, HEs, central complex, LAN1, LAN2, MAN), except for the OGT. Number and position of the 5HT-ir neurons and their immunoreactive domains in the brain neuropil will be described in detail in the following sections and are summarized in Figure 7.

#### Protocerebrum

Approximately 79–80 5HT-ir neurons are distributed in all soma clusters of the protocerebral brain hemisphere (Figure 5A). In soma cluster A, around 38–41 cells are arranged in two groups (sA1; sA2; Figure 5A). Group sA1 is localized more dorsally in soma cluster A near the midline and includes 13 5HT-ir cells in both hemispheres, the neurites of which project posteriorly into the protocerebral neuropil (Figure 5B). Immediately after entering the neuropil, the projections of sA1 converge with neurites of a 5HT-ir neuropilar domain (sd1) positioned laterally to sA1 (Figure 5B). Two neurite bundles emerge from this point of convergence: one branch extends anteriorly, where some neurites decussate with axons of the other brain hemisphere (Figure 5C). After this decussation, the neurites project anteriorly (Figure 5B, C, red arrows). The other branch from this area of convergence extends towards the midline, forming a diffuse net of decussations before it extends posteriorly towards the 5HT-ir domain of an unpaired midline structure in the center of the protocerebrum (sd2; Figure 5D). Furthermore, a faint labeling of 5HT-ir neurites can be observed in the HEs. Neurites from sd1 enter anteromedially, arborizing in the HE (Figure 5B). The domain sd2 is positioned close to two spherical domains composed of a dense network of 5HT-ir neurites (sd3; Figure 5D). Moreover, sd2 is posteriorly connected with three small 5HT-ir domains (sd4-6) on each hemisphere of the brain via extremely fine 5HT-ir projections (Figure 5D). The domains sd4-6 are interconnected by 5HT-ir neurites, which decussate in the midline, forming an arcuate structure (Figure 5D). More ventrally, numerous 5HT-ir neurites extend from the unpaired midline domain sd2 anteriorly, forming another unpaired midline domain sd7 (Figure 5E). Neurites of sd7 extend further anteriorly and terminate in a bilaterally symmetric 5HT-ir domain sd8 (Figure 5E).

At a more ventrolateral position of sA1, around 25–28 5HT-ir neurons send neurites more laterally into the protocerebrum (sA2; Figures 3A and 5A, F). These neurons project anteriorly into the protocerebrum forming several commissures. After crossing the midline, one of these decussations innervates a 5HT-ir domain in a lateral position of the protocerebrum (sd9; Figure 5E). Another 5HT-ir domain (sd10) is found in close vicinity directly ventral to sd9. The domain sd10 is connected by a conspicuous commissure, with its counterpart in the opposite hemisphere of the brain (Figure 5F).

We counted 39–41 5HT-ir cells in soma cluster B. As in soma cluster A, these neurons can be divided into two groups (sB1 and sB2; Figures 2 A-D and 5A). Group sB1 is composed of eight cells in each hemisphere, which project anterolaterally into the 5HT-ir domain sd9 of the protocerebral neuropil (Figure 5B).

The lateral group sB2 consists of 31–33 somata and projects neurites on two different paths into the protocerebral neuropil (Figures 2B-D and 5A, G). Both neurite bundles converge anterior to the HEs. From this convergence, a neurite bundle loops back laterally and terminates in a 5HT-ir domain of bulbous appearance (sd9; Figure 5G). Another neurite bundle passes this convergence, connecting sd4 and the lateral sd11 (Figure 5E).

Due to the conspicuous pattern of 5HT-IR in the protocerebrum, we conclude, that sd2 and sd3 correspond to structures within the central body. The 5HT-ir domains sd4-6 resemble subcompartments of the protocerebral bridge, which is connected via very fine W-, X-, Y-, Z-tracts to the central body. Furthermore, due to its connecting tract to the central body, sd9 represents the lateral accessory lobe. Thus, these 5HT-ir domains and connecting fibers are part of the central complex in Remipedia.

#### Deutocerebrum

In the deutocerebrum, we observed about 36–38 5HT-ir neurons in the soma clusters D and E, whereas soma cluster C does not contain any 5HT-ir cells (Figures 3 and 6A).

In soma cluster E, two 5HT-ir cell bodies (sE1) can be observed in one hemisphere, projecting their neurites in a loop dorsolaterally towards the LAN2 (Figures 3C, D and 6A, C). From the main neurite bundle, projections branch off to innervate two compartments (sd12 and sd13) of LAN2 with a fine 5HT-ir network (Figure 6A, C). The main neurite bundle continues ventromedially crossing the midline and forming a decussation with its counterpart from the other brain hemisphere. Near the midline, another neuropil, which seems to be associated with the MAN, is innervated by neurites of this commissure (sd14; Figure 6D).

A group of 22–24 5HT-ir neurons located in soma cluster D (sD1) innervates parts of the MAN by a short medial projection (sd15; Figures 3A, B and 6A, E, E’). The domain sd15 is interconnected to other 5HT-ir domains in the MAN (Figure 6E, E’).

Another group of 5HT-ir neurons is situated in soma cluster D. Eight 5HT-ir neurons in both hemispheres project anteriorly to the ONs (sD2; Figures 3A, B and 6A, E, E’). Entering the core of the ONs parallel to the OGT, the projections ramify and extend into the different sublobes of the ON. Within these sublobes, all olfactory glomeruli seem to be innervated by 5HT-ir neurites (Figure 6F).

#### Tritocerebrum

In soma cluster E, about 2–8 5HT-ir neurons are situated anteromedially to the tritocerebral neuropil (sE2; Figures 3C and 6B). The neurites of these neurons in sE2 project into the tritocerebral neuropil and terminate in a diffuse plexus of 5HT-ir neurites (sd16; Figure 6B).

### Brain anatomy and serotonin immunoreactivity of *Godzilliognomus frondosus*

**Figure 8:**
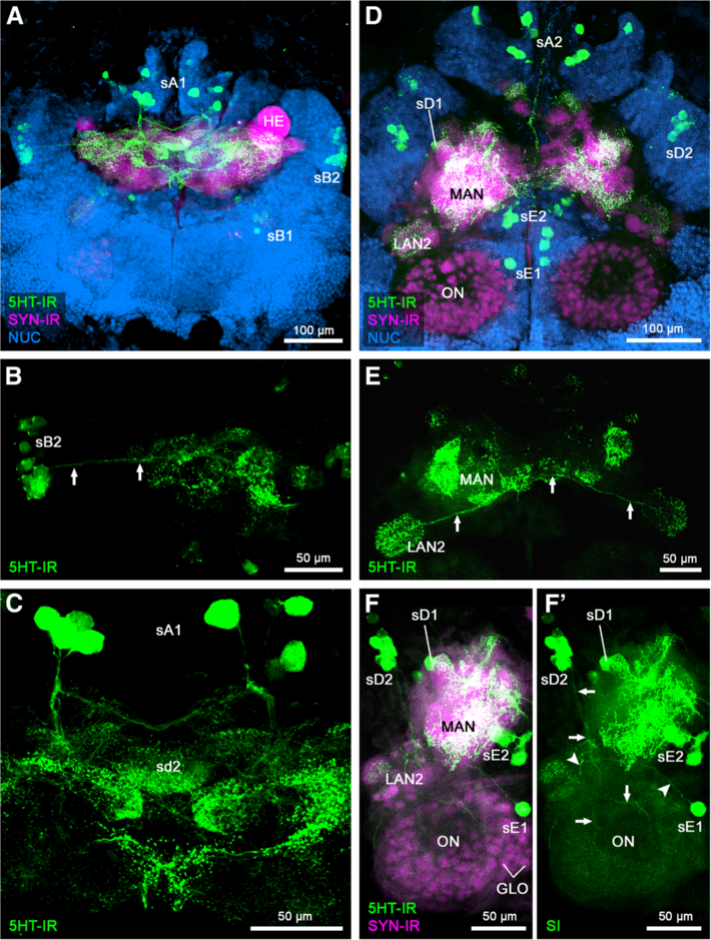
**Serotonin immunoreactivity in the brain of* Godzilliognomus frondosus. *** (**A**-**F**’) Maximum projections of horizontal vibratome sections (100 μm) triple labeled for serotonin immunoreactivity (5HT-IR; green), synapsin immunoreactivity (SYN-IR; magenta) and nuclear marker (NUC; blue). (A, D) Overview of the protocerebrum (**A**) and deutocerebum (**D**) of *G. frondosus* showing the positions of 5HT-ir cell groups sA1, sA2, sB1, sB2, sD1, sD2, sE1, sE2. In the protocerebrum, sB2 (**B**) and sA1 (**C**) show the same projection patterns as in *Speleonectes tulumensis* (arrows; compare to Figure 5). A conspicuous unpaired midline domain is situated in the center of the protocerebrum in the same position as in *S. tulumensis* (sd 2; C). (**E**) In the deutocerebral paired lateral antenna 1 neuropil 2 (LAN2) strong 5HT-IR can be detected and are interconnected by a 5HT-ir commissure (arrows) originating from a single 5HT-ir neuron (sE1) (arrowheads in F’). (**F**, **F**’) Additionally, neurites from eight 5HT-ir neurons (sD2) grow out anteriorly into the core of the olfactory neuropils (ONs), where they ramify to a dense meshwork as in *S. tulumensis* (arrows; compare to Figure 5). Abbreviations: GLO: olfactory glomerulus; HE: hemiellipsoid body; LAN2: lateral antenna 1 neuropil 2; MAN: medial antenna 1 neuropil; ON: olfactory neuropil; sA1, sA2, sB1, sB2, sD1, sD2, sE1, sE2: 5HT-ir cell groups; sd1-9, sd11-15: 5HT-ir domains.

In comparison to the results of *Speleonectes tulumensis*, the 5HT-IR appears relatively indistinct and unspecific. This might be due to the difficulties of dissecting the specimens at the collection site so that an adequate fixation could not be achieved. Nevertheless, in the brain of *Godzilliognomus frondosus* (Godzilliidae), the 5HT-IR exhibits the same main characters as in *S. tulumensis* (Figure 8), and all clusters of 5HT-ir somata can be detected in *G. frondosus*. Differences were confined to the number of 5HT-ir neurons. The brain of *G. frondosus* comprises only 57 or 64 5HT-ir cell bodies in each hemisphere, which is approximately half of the 5HT-ir neurons of *S. tulumensis*.

The soma clusters A and B are associated with the protocerebrum and host the 5HT-ir neurons sA1, sA2, sB1 and sB2, which extend their neurites into the protocerebral neuropil (Figure 8A-C). While these neurites enter the neuropil in the same way as in *S. tulumensis*, their projections could not be followed in as much detail. Due to the indistinct 5HT-IR, a comprehensive description of all the 5HT-ir domains is not possible. Nevertheless, at least one conspicuous 5HT-ir domain can be recognized. Because of the shape and position within the neuropil, we interpret the unpaired midline neuropil in the center of the protocerebrum as sd2 (Figure 8A, C; compare to Figure 5D, E).

In soma clusters D and E, which supply the deutocerebral neuropils, the 5HT-ir groups sD1, sD2, sE1 and sE2 are distributed in the same manner as in *S. tulumensis* (Figure 8D-F). The cell group sD1 consists of 7–8 5HT-ir neurons and sends out a neurite bundle anteriorly into the core of the ONs, where it splits to innervate the olfactory glomeruli (Figure 8F, F’). For *G. frondosus*, we counted 291 and 311 olfactory glomeruli in the two ONs.

As specified for *S. tulumensis*, a 5HT-ir neuron sE1 projects from soma cluster E positioned near the midline laterally to the LAN2 (Figure 8F, F’). However, a subdivision into two 5HT-ir domains cannot be observed in *G. frondosus.* From here, the neurite bundle loops medially, crossing the midline and forming a decussation with its counterpart from the other brain hemisphere (Figure 8E). The projections from sD2 and sE2 cannot be traced in detail.

## Discussion

Due to the difficulties in collecting Remipedia, data on their brain anatomy so far were limited to the studies by Fanenbruck et al. [15,16]. Fortunately, we were able to obtain three specimens of Remipedia preserved for immunocytochemistry. In this study we present the first analysis of the brain anatomy of Remipedia based on immunocytochemical methods and laser-scanning microscopy. These methods provide a much clearer resolution of neuropils, tracts, and single soma clusters than previously available [15,16], and therefore allow for a more detailed description of neuroanatomical structures. Besides new insights concerning the general brain anatomy, our comprehensive description of the 5HT-IR in the brain resolves individually identifiable cell groups in Remipedia.

The neuroanatomical differences we detected in *S. tulumensis* and *G. frondosus* might reflect a phylogenetic divergence between the representatives of two remipede families, Speleonectidae and Godzilliidae. However, it is equally conceivable that the differences are the result of the histological fixation procedure under field conditions. Furthermore, a circadian fluctuation of the serotonin level as known from other crustaceans (see below) could be responsible for the differences of 5HT-IR in both species.

Our results have led to some differences in the interpretation of the brain anatomy of Remipedia compared to Fanenbruck et al. [15,16]. These authors described a small unpaired transverse midline neuropil in the posterior proximity to the chiasm of the OGT that they identified as the central body (Figure 1D). Fanenbruck et al. characterized a neuropilar structure that most likely corresponds to the protocerebral bridge anterior to the chiasm of the OGT. In this position we identified the 5HT-ir midline domain sd2. Due to the branching pattern of the 5HT-IR in this domain and its medial position in the protocerebrum we interpret sd2 as the central body, which is positioned anteriorly to the chiasm of the OGT (Figures 4E and 5D). Furthermore, the 5HT-ir domains 4–6 are located within the protocerebral bridge, and sd9 most likely corresponds to the lateral accessory lobe of the central complex (Figure 5E).

Fanenbruck et al. [15,16] described a small glomerular neuropil located anterior to the ON as olfactory satellite neuropil. We detected two small domains (sd12 and sd13), both receiving input from antenna 1. These domains seem to belong to the same neuropil that is innervated by projections of two 5HT-ir neurons (sE1; Figure 6C). Because of the connection to antenna 1, we conclude that this structure resembles compartments of the LAN2, and that it is not an olfactory satellite neuropil, as previously suggested [15,16].

### Comparative Neuroanatomy

The dominant neuropils in the deutocerebrum of Remipedia are the ONs. We counted about 300 spherical olfactory glomeruli for *Godzilliognomus frondosus* and approximately 375 olfactory glomeruli for *Speleonectes tulumensis*. In both species, all of these olfactory glomeruli are innervated by approximately eight 5HT-ir neurons from the deutocerebral soma cluster D (Figures 6E, E’ and 8F, F’). An innervation of the olfactory glomeruli by 5HT-ir neurons is known from a variety of Pterygota, Collembola, Decapoda (Malacostraca) and Cephalocarida; however, the number and branching patterns differ between species [33-36]. All representatives of Pterygota studied to date show one to three 5HT-ir neurons innervating the antennal lobe, with additional projections into the protocerebrum [34]. In Collembola, one centrifugal neuron situated in the subesophageal ganglion innervates one or four olfactory glomeruli of the antennal lobe, but never all olfactory glomeruli [36]. In the studied Crustacea that possess 5HT-ir neurons associated with the ONs, these neurons always innervate the ipsilateral ON and lack projections into the protocerebrum. The most comprehensive information is available for Decapoda (reviews [35,37]). Two different types of 5HT-ir olfactory interneurons were classified by Johansson [38]: the giant interneurons and the smaller globuli cells. Apart from the identifiable dorsal giant interneuron, several other large 5HT-ir neurons were observed with species-specific variation in number, e.g., two to four pairs in spiny lobsters and crayfish per brain hemisphere (e.g., [39-41]). Furthermore, high numbers of 5HT-ir globuli-type cells, which are much smaller than the giant interneurons, were documented for a variety of decapods, reaching numbers up to several hundred cells [41]. Several authors showed that the immunoreactivity of some 5HT-ir neurons is restricted to certain subregions within each olfactory glomerulus, e.g., the cap and base in the American lobster [42] or the subcap and base in the spiny lobster [41]. Additionally, an interconnection by 5HT-ir neurites between neighboring olfactory glomeruli has not been observed [41]. Data for non-decapod Malacostraca concerning 5HT-IR are sparse. Moreau et al. [43] detected diffuse staining in the olfactory glomeruli in Mysidacea, with the possible origin in two cell bodies adjacent to the ON. In Amphipoda, a giant tritocerebral 5HT-ir neuron innervates most parts of the brain, including the ONs [44].

Stegner and Richter have recently investigated the brain anatomy of a representative of Cephalocarida using immunocytochemistry [33]. This group takes on an important role in the discussion of the phylogenetic position of Remipedia, because a close relationship to Remipedia has been proposed by several molecular studies [18-20]; [45-47]. In *Hutchinsoniella macracantha*, the only species of Cephalocarida for which data on brain anatomy exist to date, the olfactory glomeruli are arranged in seven vertical columns [33]. Two 5HT-ir neurons innervate the ONs. However, one of the seven columns is not innervated by 5HT-ir neurites. In the other six, the innervation is concentrated on the inner margin of each olfactory glomerulus. Adjacent olfactory glomeruli, the shape of which is very different to that of other Mandibulata as will be discussed below, are interconnected by 5HT-ir neurites [33].

Besides Cephalocarida, only a few non-decapod Crustacea have been investigated for 5HT-IR in the deutocerebrum. These include Branchiopoda, Copepoda and Mystacocarida, all of which lack 5HT-ir interneurons [28,30,31,48].

In contrast to Decapoda or Cephalocarida, each olfactory glomerulus in Remipedia seems to be innervated relatively homogenously by 5HT-ir neurites that are not restricted to certain subregions within the olfactory glomeruli (Figure 6F). Serotonin immunoreactive neurites, directly linking adjacent olfactory glomeruli as in Cephalocarida, are absent. Remipedia do not possess any projections from the 5HT-ir cells into the protocerebrum as do the corresponding neurons in many insect taxa [34]. In summary, Malacostraca, Cephalocarida, Hexapoda and Remipedia show 5HT-ir innervation of the glomeruli in the ON, but the number, arrangement and innervation pattern of the 5HT-ir neurons differ among these taxa. At this point, we cannot decide whether the 5HT-ir innervation of the ONs is part of the ground pattern in Tetraconata or a functional convergence, an issue that has recently also been discussed with regard to 5HT-IR of chelicerate chemosensory pathways [49]. A basic serotonergic innervation may be a feature that characterizes all neuromeres in the central nervous system of Euarthropoda, so that the deutocerebral serotonergic system may just be a modification of such a basic supply [49].

### Organization of the olfactory neuropils

Within the glomerular organisation of the ONs known from numerous arthropod taxa, differences in number, shape and arrangement are evident. As in Remipedia, spherical olfactory glomeruli occur in hexapods like Diplura, Zygentoma [50], Collembola [36] and most Pterygota [35], but also in malacostracans, such as Stomatopoda [51], Leptostraca (Kenning M, Müller CHG, Wirkner CS, Harzsch S, unpublished results; [24]) and marine Isopoda [52]. Many decapod crustaceans possess wedge-shaped or cone-shaped olfactory glomeruli, while Archaeognatha seem to be the only hexapod taxon exhibiting elongated olfactory glomeruli (review [35] and references therein; [53]). Even though the detailed shape of cephalocaridan olfactory glomeruli was difficult to resolve by immunocytochemical methods, it is certainly not spherical [33]. Studies on Branchiopoda, Copepoda and Mystacocarida revealed that these taxa do not possess ONs and consequently do not have any glomerular structures in the deutocerebrum [30,31,48].

Olfactory glomeruli of spherical shape were proposed to be part of the tetraconate ground pattern [35]. Recently, spherical olfactory glomeruli were described in some chilopods [54], which led to the suggestion that spherical olfactory glomeruli might be part of the mandibulate ground pattern. In this view, elongated, wedge- or cone-shaped olfactory glomeruli represent a derived attribute. However, because elongated or drop-shaped olfactory glomeruli also occur in certain representatives of Chilopoda [54], it is as yet unclear which shape represents the plesiomorphic character state as part of the mandibulate ground pattern.

The glomeruli in the ONs of Remipedia are arranged in at least four sublobes that are clearly separated from each other (Figure 6F). From the center of the ONs, neurite tracts extend into these different sublobes. Within the sublobes, the olfactory glomeruli are arranged like clusters of grapes. Subdivision of the ONs into several lobes is also known from a certain subgroup of Decapoda, the Coenobitidae (terrestrial hermit crabs). However, in contrast to Remipedia, their wedge-shaped olfactory glomeruli are arranged radially around the outer margin of three sublobes and the sublobes are not as clearly separated as those in the Remipedia (e.g. [29,32]). In conclusion, the anatomy of the ONs in Remipedia represents a special and unique way of arranging olfactory glomeruli without any close resemblance to the topology of other Crustacea or Hexapoda.

### Connections between Deuto- and Protocerebrum

Distinct fiber tracts, the olfactory globular tracts (OGTs), that connect the deutocerebral ONs to protocerebral structures were reported for Cephalocarida [33,55], decapod Malacostraca [25,56], Copepoda [57] and Remipedia ([15,16], this study). In Hexapoda, one or more tracts with equivalent topology, here called antennocerebral tracts, project from the antennal lobe to specific protocerebral neuropils (reviews [35,58]). Protocerebral targets of the antennocerebral tracts in the Hexapoda are the mushroom bodies. The OGTs target the multilobed complex in the Cephalocarida, the hemiellipsoid bodies (HEs) and the medulla terminalis (termed lateral protocerebrum; [25,59]) in Malacostraca, and in Remipedia the HEs. In the majority of studied Malacostraca and in Remipedia, the paired branches of the OGT form a characteristic chiasm in the center of the protocerebrum [15,16,25]. Recently, a chiasm of the OGT similar to that in Remipedia and Malacostraca was reported for the copepod *Tigriopus californicus*[57].

In Remipedia, we detected a small neuropil (Figure 4D) that lies near the HE and is connected to it by 5HT-ir neurites. From a 5HT-ir domain sd11 in this neuropil, some 5HT-ir axons project into the protocereral bridge (Figures 5E and 7). This resembles the situation in the decapod *Cherax destructor*[40]. Based on the connectivity to the HE and the pattern of 5HT-IR, we interpret this neuropil as a medulla terminalis, which together with the HE forms the lateral protocerebrum. In Mandibulata, the lateral protocerebrum is associated with the optic neuropils. The four retinotopic optic neuropils, lamina, medulla, lobula plate and lobula [60], which characterize the central visual pathway associated with the compound eyes of malacostracan Crustacea and Pterygota, are absent in Remipedia [16]. Therefore, the medulla terminalis receives no input from the optic neuropils, and this might explain the reduction of the medulla terminalis in Remipedia.

Serotonin immunoreactivity in the neuropil of the HE has been detected in the decapod *Procambarus clarkii* and *Orconectes rusticus*[61], but not in *Cherax destructor*[40] and *Pacifasticus leniusculus*[62]. In Remipedia, we observed 5HT-IR; however, contrary to Decapoda, this innervation does not originate from the medulla terminalis, but from a neuropil near the median protocerebrum (Figure 5B). In contrast to Decapoda, [61] we did not find a division of the HE into two distinct neuropils and an innervation of the medulla terminalis by the OGT. Furthermore, the HE is not organized in distinct layers as in some Decapoda and Stomatopoda [29,61,63]. In other Decapoda, the HEs do not show layers but microglomeruli [64], indicating distinct structural variations of the HEs in Malacostraca. In spite of these structural differences, we suggest that the HEs of Remipedia and Malacostraca are homologous based on their protocerebral position, spherical shape and the connectivity to the deutocerebral ONs by the OGT.

### Unpaired midline neuropils of the Protocerebrum

Unpaired midline neuropils are a common feature in the protocerebrum of e.g. Chelicerata, Onychophora [26,27,65,66], Myriapoda [54], Hexapoda and in crustacean species belonging to Malacostraca [26,67], Remipedia ([15,16], this study) and Branchiopoda [28]. In general, the central complex of Tetraconata consists of the unpaired central body, the unpaired protocerebral bridge, and the paired lateral accessory lobes (see neuroanatomical glossary [68] and [69]). Utting et al. [67] performed a detailed study of the central complex of the crayfish and revealed a number of similarities in the overall architecture, neuronal projections and immunoreactivity of neurons, and suggested a possible homology of this complex to that of insects.

Serotonin immunoreactivity in the central complex has been studied in a variety of Hexapoda and Crustacea. In all studied taxa, neuropils of the central complex show 5HT-IR which originates from 5HT-ir neurons in soma clusters situated lateral and posterior to the protocerebral neuropil. In Remipedia, the labeling against serotonin reveals a conspicuous unpaired midline domain sd2 in the center of the protocerebrum (Figure 5E). Furthermore, sd2 is interconnected by fine neurites with an arcuate structure consisting of the paired sd4-6 and by a thick neurite bundle to the laterally positioned sd9. Because of the location and connectivity between these neuropils, which appears similar to that present in other Crustacea and Hexapoda, we consider these structures to be the central complex, comprising the central body (sd2), the protocerebral bridge (sd4-6) and the lateral accessory lobes (sd9). However, we could not find a separation of the central body in an upper and lower division and, similar to all other crustacean taxa, we did not detect noduli as in insects [26].

In both Crustacea and Hexapoda, 5HT-ir neurons innervate the lateral accessory lobes and then form a commissure near the central body to the contralateral brain hemisphere [67,70,71]. Similar neurons are positioned in soma cluster B in Remipedia (sB1; Figure 5B), projecting into the lateral accessory lobes. Whether these neurons contribute to a protocerebral commissure remains unclear. In addition, Remipedia possess various 5HT-ir neurons in soma cluster A, associated with the central complex, especially the central body (Figure 5B). The 5HT-ir neurons sA1 thus may correspond to the central body neurons 1 and 2 (CBN1 and CBN2) in crayfish [67]. Comparable cells are present in Branchiopoda and Insecta [71,72]. In locusts and crayfish, the protocerebral bridge and the central body are connected via four tracts in each brain hemisphere, the W-, X-, Y-, Z-tracts, containing 5HT-ir neurites [66,71]. Corresponding neurites have been found in Remipedia (yellow arrowheads in Figure 5D), however, they do not seem to form a chiasm before entering the central body as it is present in locusts. This chiasm is also missing in crayfish [67]. In Remipedia, the tracts connecting protocerebral bridge and central body could be identified only with 5HT-IR. In conclusion, we provide first evidence of W-, X-, Y-, Z-tracts in Remipedia, but further studies are desirable to confirm homology.

The unpaired sd2 in Remipedia receives input from axons of the 5HT-ir neurons sA2 that cross the midline before entering sd2 anteriorly. In Cephalocarida, Malacostraca and Hexapoda corresponding 5HT-ir neurons are situated anteriorly to the central complex. The neurites of these cells decussate before innervating parts of the central body [33,40,71]. Unpaired 5HT-ir domains in the center of the protocerebrum have also been described for Cephalocarida and Mystacocarida [30,33], but other compartments of the central body are missing in these species. Therefore, the described distribution and innervation pattern of the central complex by 5HT-ir neurons seems to be a shared feature in Remipedia, Malacostraca and Hexapoda.

### Functional considerations

In Arthropoda, the neuromodulator and neurohormone serotonin is involved in a variety of physiological processes, and a circadian fluctuation of serotonin levels has been observed in a number of crustacean taxa [73,74]. This could be an explanation for the differences in the results of 5HT-IR in *Speleonectes tulumensis* and *Godzilliognomus frondosus*. In Decapoda, it was shown that the serotonin level fluctuates in a bimodal or trimodal manner, indicating circadian control [75,76]. Sandeman et al. [40] revealed a 5HT-ir connectivity of the protocerebral bridge, the medulla terminalis and the sinus gland in the eyestalk of *Cherax destructor*. Removal of this gland in *Procambarus clarkii* caused changes in the circadian rhythm [77,78]. Remipedia show similar 5HT-ir projections between the protocerebral bridge and the medulla terminalis.

## Conclusions

Remipedia and Malacostraca share several characters in their general brain organization that support a sister group relationship between both taxa. The most striking synapomorphy of Remipedia and Malacostraca is an OGT with a chiasm connecting the ONs with the lateral protocerebrum and the presence of HEs. Furthermore, the distribution of 5HT-IR in the protocerebum corresponds to the innervation of the central complex of Malacostraca and Insecta. In this context, we found first evidence for W-, X-, Y-, Z-tracts in Remipedia, a feature that unites Remipedia, Malacostraca and Hexapoda. Because the 5HT-ir neurites in the W-, X-, Y-, Z-tracts do not form a chiasm before entering the central body as in insects, the remipedian pattern resembles more the situation as in Malacostraca.

However, more detailed comparisons reveal some unique and taxon-specific architectural aspects of the remipede brain. For example, the study of the glomerular ONs reveals that the number and patterning of the 5HT-ir interneurons of Remipedia differs from that of all other Crustacea and Hexapoda.

In Remipedia, 5HT-ir interneurons innervate the ONs and an unpaired midline neuropil in the center of the protocerebrum. These features have been described for a variety of crustacean and hexapod species, including taxa like Collembola and Mystacocarida, which are considered to be basal representatives. Thus, in addition to a glomerular organization of the ONs, these features might be part of the ground pattern of Mandibulata or a functional convergence.

A growing number of phylogenetic studies based on molecular data suggest Cephalocarida to be the sister group of Remipedia [18-20,45-47]. In contrast, our comparative neuroanatomical study on the brain does not provide support for such a close relationship.

## Methods

### Collection, fixation procedures and dissection of specimens

Two individuals of *Speleonectes tulumensis* (Speleonectidae) were collected in the Cenote Crustacea on the Yucatan Peninsula, Mexico. Additionally, one specimen of *Godzilliognomus frondosus* (Godzilliidae) was obtained from Sawmill Sink on Abaco Island, Bahamas. Animals were fixed in 4% paraformaldehyde dissolved in phosphate-buffered saline (PBS, 10 mM sodium phosphate, 150 mM sodium chloride, pH 7.4) immediately after collecting. In order to achieve a better penetration of the tissue by the fixative, the head region, together with some trunk segments, was separated and head appendages were cut off. After shipment, tissue was rinsed four times in PBS for at least 30 min each and stored in PBS at 4°C with 0.5% sodium azide until use.

Horizontal and frontal sections were cut by a vibratome (Hyrax V 50, Zeiss). For better connection of tissue and agarose, specimens were incubated shortly in Poly-L-Lysin and then embedded in 4% agarose dissolved in aqua dest. at approximately 50°C. After cooling to room temperature, the trimmed blocks were sectioned into series of 50–100 μm thin sections.

### Immunocytochemistry

All steps of immunocytochemistry were performed on a shaker with smooth agitation and at room temperature if not otherwise stated. Sections were permeabilized for 45 min with 0.3% Saponin in PBS containing 0.2% Triton X-100 (PBS-TX 0.2%), washed four times for 30 min each in PBS-TX 0.2%, and afterwards incubated for 3 h in blocking solution (5% normal horse serum (Vector) in PBS-TX 0.2%). In the next step, sections were incubated for 48 h at 4°C with primary antibodies diluted in blocking solution. The following antisera were used: the polyclonal antibody rabbit anti-serotonin (Sigma, dilution 1:2000) and the monoclonal antibodies mouse anti-synapsin “SYNORF1” (DSHB University of Iowa, 1:30) [79] and mouse anti-acetylated α-tubulin (Sigma, 1:500). Subsequently, the tissue was rinsed for 2 h in four steps in PBS-TX 0.2% and incubated in a mix of the secondary antibodies goat anti-rabbit Cy3-conjugated (Jackson Immuno Research Laboratories) and goat anti-mouse Alexa Fluor 488-conjugated (Molecular Probes) in a dilution of 1:250 each plus 4^′^6-diamidine-2-phenylidole-dihydrochloride (DAPI) for counterstaining the nuclei (1 μg/ml) in blocking solution overnight at 4°C. Finally, all tissues were washed for 2 h in several changes with PBS-TX 0.2% and PBS (last step) and mounted on glass slides in Mowiol.

For the three specimens the following combinations of markers were performed:

*Speleonectes tulumensis* specimen 1: serotonin, acetylated α-tubulin, nuclei

*Speleonectes tulumesis* specimen 2: serotonin, synapsin, nuclei

*Godzilliognomus frondosus*: serotonin, synapsin, nuclei

The following abbreviations and color-codes identify the markers in the figures:

5HT-IR: serotonin immunoreactivity (green)

TUB-IR: acetylated α-tubulin (magenta)

SYN-IR: synapsin immunoreactivity (magenta)

NUC: counterstaining of nuclei with DAPI (blue)

### Antibody specificity

#### Serotonin

For serotonin labeling, a polyclonal rabbit antiserum raised against a serotonin creatinine sulfate complex conjugated to bovine serum albumin as the immunogen (Sigma, cat. no. S5545, lot no. 108 K4868) was used. Because of the difficulty of collecting specimens of Remipedia (see Background), no experiments concerning the specificity of antisera could be conducted. However, 5HT-IR was investigated in numerous invertebrate phyla [80], including studies on all kinds of Arthropoda (Chelicerata and Myriapoda: e.g., [49,81]; Hexapoda: e.g., [34,71,82,83]; Crustacea: e.g., [30,31,33,84]). Due to this wide distribution in the nervous systems of all kinds of animals and the similar distribution pattern (e.g.,[34,84]) serotonin as a neurotransmitter seems highly conserved in evolution [80]. This leads to the suggestion that the antibody also labels serotonin in Remipedia.

#### Acetylated α -tubulin

In order to describe the general morphology of the remipede brain, we used a monoclonal antibody raised against acetylated α-tubulin from the sea urchin *Strongylocentrotus purpuratus* (Sigma, cat. no. T6793, lot no. 059 K4823, clone 6-11B-1). This antibody reacts with acetylated α-tubulin over a wide range of organisms such as plant, human, pig, monkey, invertebrates, hamster, bovine, chicken, rat, frog, protista and mouse (see datasheet manufacturer) and was utilized in numerous studies on the nervous system of the major crustacean taxa except Remipedia (e.g., Branchiopoda: [31]; Cephalocarida: [33]; Malacostraca: [85]; Maxillopoda: [30]). Thus, the recognized epitope seems to be highly conserved across life forms, which leads to the suggestion that the antiserum labels acetylated α-tubulin also in Remipedia.

#### Synapsin

The synapsin antiserum is a monoclonal mouse anti-*Drosophila* synapsin antibody (“SYNORF1”, Developmental Studies Hybridoma Bank, University of Iowa, 1:30) raised against a *Drosophila* GST-synapsin fusion protein. In western blots of head homogenates of *Drosophila*, at least four isoforms of synapsin are recognized by this antiserum [79]. Harzsch and Hansson compared western blot results of brain tissues of *Drosophila* and the crustacean *Coenobita* (Anomura) [29]. In this study, the antiserum stained identical bands in both species, which suggests that the epitope for SYNORF 1 is strongly conserved between *Drosophila* and *Coenobita*. In addition, in numerous studies, the synapsin antibody stained neuropil structures over a wide range of arthropod taxa, for example Crustacea (Branchiopoda: [28,86]; Malacostraca: [29,85,87]), Hexapoda (e.g. [36,88]), Chilopoda [89] and the spider *Cupiennius*[90]. The similar staining pattern of synaptic neuropils leads to the suggestion that the synapsin antibody reacts with a highly conserved epitope, and that this antiserum in fact labels synaptic neuropils in the remipede brain.

### Image acquisition and processing

Confocal images and z-stacks were taken with a Leica TCS SP5 confocal laser-scanning microscope using Leica LAS AF software. Every physical section was scanned, resulting in stacks of optical sections with 0.5 μm thickness. One preparation labeled against serotonin, acetylated α-tubulin and nuclei consisted of eight physical sections and in total 247 optical sections. This image stack was aligned with the Amira 3D reconstruction software in order to receive a 3D virtual stack of the remipede brain. Z-series were processed with NIH ImageJ, v. 1.44 (Rasband, W.S., ImageJ, U.S. National Institutes of Health, Bethesda, MD, http://rsb.info.nih.gov/ij/), producing depth coded images and merging channels. The quality was enhanced by adjusting brightness and contrast if necessary and photographs were arranged using Adobe Photoshop 6.0 (San Jose, CA).

### Counting of olfactory glomeruli

The number of olfactory glomeruli was manually counted for each ON in the specimens with synapsin-labeling (one specimen of *Speleonectes tulumensis* and *Godzilliognomus frondosus*, respectively). To avoid double counting, each olfactory glomerulus was marked using ImageJ including the Cell counter plugin (De Vos, K., University of Sheffield).

### Nomenclature

We use the neuroanatomical standard nomenclature as suggested by Richter et al. [68] and compared the labeled structures to the brain atlas of Remipedia by Fanenbruck et al. [15,16]. These authors related the brain structures to the neuroanatomy of Decapoda [25,56].

## Competing interests

The authors declare no competing interests.

## Authors’ contributions

TMI conducted the sampling and fixation of specimens. TS carried out the vibratom sectioning, the immunocytochemical experiments and the confocal laser-scanning microscopy. TS drafted the first version of the manuscript and all other authors assisted in drafting the manuscript. All authors read and approved the final manuscript.
